# Biopsy-derived oral keratinocytes – A model to potentially test for oral mucosa radiation sensitivity

**DOI:** 10.1016/j.ctro.2022.03.007

**Published:** 2022-03-16

**Authors:** A.R. Thomsen, C. Aldrian, B. Luka, S. Hornhardt, M. Gomolka, S. Moertl, J. Hess, H. Zitzelsberger, T. Heider, N. Schlueter, S. Rau, B. Monroy Ordonez, H. Schäfer, G. Rücker, M. Henke

**Affiliations:** a Department of Radiation Oncology, University Medical Center, University of Freiburg, Freiburg/Breisgau, Germany; b German Cancer Consortium (DKTK) Partner Site Freiburg, German Cancer Research Center (dkfz), Heidelberg, Germany; c Division for Cariology, Department of Operative Dentistry and Periodontology, Center for Dental Medicine, Medical Center - University of Freiburg, Faculty of Medicine, University of Freiburg, Germany; d Federal Office for Radiation Protection, Ingolstädter Landstr. 1, 85764 Oberschleißheim, Germany; e Research Unit Radiation Cytogenetics, Helmholtz Zentrum München, German Research Center for Environmental Health GmbH, Neuherberg, Germany; f Clinical Cooperation Group “Personalized Radiotherapy in Head and Neck Cancer”, Helmholtz Zentrum München, German Research Center for Environmental Health GmbH, Neuherberg, Germany; g Department of Radiation Oncology, University Hospital, LMU Munich, Munich, Germany; h Institute for Medical Biometry and Statistics, Medical Center – University of Freiburg, Germany

## Abstract

**Purpose:**

To establish stable *in vitro* growth of keratinocytes from very small biopsy specimens and successfully apply new test systems to determine their radiosensitivity.

**Materials and Methods:**

Oral mucosa biopsies (diameter: 1.7 mm) from 15 subjects were immobilized with custom-made cups onto culture plates. Outgrowing cells were tested for cytokeratin 5/14 and Ki67, expanded, radiated at different doses, and seeded onto circumscribed areas before being allowed to spread centrifugally. In this newly developed spreading assay, cell-covered areas were measured by image analysis. For statistical analysis, a linear mixed regression model was used; additionally, results were correlated to the radiation dose applied. Colony forming efficiency (CFE) was used to validate the results. DNA damage repair was analysed by gammaH2AX and 53BP1 foci quantification using immunofluorescence microscopy 24 h and 96 h after irradiation.

**Results:**

Stable keratinocyte growth continued for up to 7 weeks in 14 biopsies. Cells spread reliably from an initial 16.6 mm^2^ up to a median of 119.2 mm^2^ (range: 54.4–290). Radiated cells spread to only 100.7 mm^2^ (2 Gy; range: 55.3–266.7); 73.2 mm^2^ (4 Gy; 15–240.4); 47 mm^2^ (6 Gy; 2–111.9), and 22.7 mm^2^ (8 Gy; 0–80). Similarly, CFE decreased from 0.223 (0 Gy) to 0.0028 (8 Gy). Using an individual donor as a random factor, cell spread correlated with CFE, where radiation dose was the main driver (decrease by 0.50, adjusted for area). Upon irradiation with 6 Gy, radiation-induced DNA damage was increased after 24 h in all samples, and even after 96 h in 5 out of 7 samples, as detected by a higher number of gammaH2AX/53BP1 foci in irradiated cells (mean 3.7 for 24 h; mean 0.6 for 96 h).

**Conclusion:**

*In vitro* propagation of keratinocytes derived from a small biopsy is feasible. Radiation impairs cellular migration and proliferation, and the newly described spreading assay allows ranking for cellular radioresistance. The keratinocyte model also supports classical functional assays such as clonogenic survival and DNA double strand repair. The clinical relevance awaits upcoming investigations.

## Highlights

• Human oral keratinocytes – the key players in radiation mucositis in head and neck cancer treatment – are established *ex vivo* from patient-derived micro-biopsies.
• Individual radiosensitivity of primary oral keratinocytes is measured by a novel assay for cellular proliferation and spreading.
• The keratinocyte model also supports classical functional assays such as clonogenic survival and DNA double strand repair.

## Introduction

While endowed with a remarkable regeneration capacity, the mucosa of the oral cavity and pharynx is very sensitive to ionizing radiation. Thus, patients undergoing radiotherapy for head and neck cancer are likely to suffer from inflammation and ulcerations, called radiation mucositis, which causes severe discomfort and can even lead to life-threatening complications [1], [2], [3]. These severe reactions of normal tissue hamper optimal tumour control by reducing the therapeutically necessary radiation dose. An accurate prediction of the radiation response of normal tissue would allow one to adapt treatment options. As yet no clinical biomarker for radiation mucositis is available [4]. One reason for the lack of biomarkers or adequate bioassays for mucosal tissue is that biomarkers or bioassays have mainly been tested in blood, but not in the target tissue where side effects occur. In addition, oral mucosa is a mainly 2-dimensional tissue that can not be assessed by metabolic imaging in a way like other tissues at risk for radiation damage, e.g. salivary glands [4]. Therefore, acquisition of oral keratinocytes from individual patients where radiation response and additional cellular properties can be identified could help to better understand the pathophysiology of radiation-associated toxicity, and guide personalized treatment.

To pursue individualized strategies in the context of radiation mucositis, it is necessary to use biopsy procedures that are as minimally invasive as possible in order to avoid delaying the onset of radiation by introducing an unnecessarily large wound in the oral mucosa. The balance between minimal invasiveness and reliable expansion of the cells obtained is sometimes difficult to achieve. Therefore, the aim of our study was to obtain cells from a punch biopsy with the smallest possible diameter that allowed optimal wound healing while providing sufficient cell material.

Here, we describe how to expand keratinocytes from micro-biopsies derived from single individuals and set up functional *ex vivo* assays measuring radiation-impaired migration and proliferation, as well as DNA damage repair. The assays were performed with defined cell lines for standardization as well as primary keratinocytes obtained from mucosal biopsies.

## Materials and Methods

### Cell lines

Three cell lines were used to standardize the spreading assay. Head and neck squamous cell carcinoma cell lines Cal33 and Cal27 were cultivated in DMEM, high glucose; GlutaMAX with pyruvate (#31966021, Gibco, Thermo Fisher Scientific, USA) supplemented with 10% FCS and 100 U/mL penicillin, 100 μg/mL streptomycin at 37 °C, 7.5% CO_2_ in humidified atmosphere. Immortalized oral keratinocyte cell line OKF6-Tert1 was cultivated in 1:1 Keratinocyte SFM (serum-free medium) (#17005–034, Life Technologies, Thermo Fisher Scientific, USA) and DMEM/F12 (1:1, #11330–32, Life Technologies, Thermo Fisher Scientific, USA) supplemented with BPE, EGF and 0.5x penicillin/streptomycin at 37 °C, 5% CO_2_, in humidified atmosphere. Cells were routinely checked for mycoplasma (MycoAlert, #LT07-318, Lonza Group, Switzerland) and authenticated by STR typing.

### Oral mucosa biopsy

All procedures were approved by the ethics committee of the University of Freiburg (vote ETK-FR 449/16, amended by vote ETK-FR 413/17). Written informed consent was provided. All personal data and biopsy specimens were pseudonymized. Oral micro-biopsy of mucosa was carried out in 8 healthy volunteers. To increase the sample size and to check for potential clinical damage we also included biopsies from 8 patients before chemo-radiation for cancer of the head and neck. Following a 60 s mouthwash with Chlorhexamed® Forte 0.2% (Chlorhexidinbis-D-Gluconate; GlaxoSmithKline, Germany), a superficial anaesthesia with Xylocain® Gel 2% (Lidocaine hydrochloride 1 H_2_O; AstraZeneca, Germany) was applied for two minutes to the vestibular mucosa of a tooth region (teeth 32 to 42). To lift tissue from the underlying periosteum, 0.1 mL of physiological saline was injected underneath the mucosa. Two biopsies were taken with a biopsy punch (outer diameter 2 mm, inner diameter 1.7 mm, Stiefel, SmithKline Beecham Ltd., UK) and transferred into DMEM/F12-medium (35 mM HEPES, 100 U/mL penicillin, 100 μg/mL streptomycin, 2 µg/mL ciprofloxacin). Biopsy sites were inspected one week after the procedure for eventual damage.

### *Ex vivo* propagation of human oral keratinocytes

Mucosa biopsies were cut into particles < 0.5 mm under a stereo microscope (Suppl. Figure 3) and transferred into 6-well cell culture plates (Costar). Explant culture was performed as described elsewhere [23], as a modification, mucosa particles were squeezed to the bottom of the well with agarose cups. This was done to improve take rate of the small mucosa explants, which otherwise are prone to floating off. Cups were manufactured by a replica moulding method to fit the plates. Briefly, a hot 2.4% agarose solution (DNA grade, SERVA) was poured into polydimethylsiloxane (PDMS) (Pricosil shore 31, Schwarzmann, Germany) moulds and covered with glass plates. Upon setting on wet ice, cups were removed from the moulds, immersed in D-PBS without Mg^++^/Ca^++^ (Invitrogen) and sterilized by UV radiation at 254 nm for 100 min. Cups were equilibrated in non-supplemented DMEM/F12 medium, placed onto tissue particles and thereafter filled with 2.5 mL Keratinocyte Growth Medium 2 (KGM 2) (#C-20111, Promocell, Germany), supplemented with 5% human serum (‘off the clot’), penicillin (25 U/mL) and streptomycin (25 μg/mL). Cells were incubated at 5% CO_2_, 37° C and 100 % humidity. When keratinocytes grew out of the tissue particles, agarose cups, with adherent soft tissue, were removed (usually on day 3–4) and medium was changed into KGM 2 without serum and without antibiotics. Medium was replaced every 3–4 days. On day 10–14, cells were detached with non-diluted Accutase^TM^ (PAA, Germany) for 4–10 min and re-seeded (10,000 cells per cm^2^) or further processed. To test resistance to radiation we used cells of passage 1 or 2.

Cryopreservation of keratinocytes was performed at passage 2 using KGM 2 medium supplemented with 20% human serum and 5% of dimethyl sulfoxide (DMSO, Sigma) at −1° C per minute using a Cryo-Box (Nunc Nalgene) up to −80° C. Vials were stored at −196° C in liquid nitrogen [5].

### Isolation of human mesenchymal stroma cells (hMSC; feeder cells)

hMSCs were derived from a bone marrow aspirate of a 46-year-old healthy male volunteer. The procedure was approved by the ethics committee of the University of Freiburg (vote ETK-FR 212/16). Cells were isolated from bone marrow particles as previously described [6] and expanded for three weeks. hMSCs were functionally validated by adipogenic, osteogenic and chondrogenic differentiation [6].

### Confirmation of keratinocyte growth

Keratinocytes were confirmed by anti-cytokeratin 5/14 binding antibody [7] (Sc-58733, Santa Cruz), detected with immunofluorescence labelled goat anti-mouse IgG (H + L) antibody, (Alexa Flour 594; A11020, life technologies, Germany) [7]. Cytoskeleton was counterstained with Alexa Flour 488 phalloidin (A12379, life technologies) and nuclei with DAPI (4′, 6-Diamidin-2-phenylindol, Sigma-Aldrich, Germany). Proliferation of cells was detected with Ki67 antibody (ab833, Abcam) and Alexa Flour 594-conjugated goat anti-rabbit IgG (H + L) secondary antibody.

### Radiation-induced DNA damage

Radiation-induced DNA damage was verified by 53BP1 / γ-H2AX immunostaining. Keratinocytes from seven individuals were grown on glass slides (SuperFrost Plus™, Fisher Scientific, Germany) in KSFM for 24 h, irradiated at 6 Gy, incubated for another 24 or 96 h, fixed with 2% formaldehyde in Dulbecco’s PBS for 15 min and stored (PBS) at 4 °C for further processing. Immunostaining of γH2AX and 53BP1 was performed as described [8]. Briefly, cells were permeabilized with 0.15% Triton X-100 in PBS 3x for 5 min. Slides were blocked with blocking solution (1% BSA (Sigma Aldrich, Germany)/0.15% glycine/PBS) and incubated with 75 µL of anti-phospho-histone H2A.X (Ser139) mouse mAb (#05–636, Merck, Germany) 1:100 and anti 53BP1 rabbit pAB (#NB100-305, Novus Biologicals, USA) 1:500 for 2 h at room temperature. After washing (5 min PBS, 10 min 0.15 % Triton X-100 in PBS, 5 min PBS and 7 min with blocking solution) slides were incubated with 75 µL of anti-mouse IgG (H + L), F(ab’)_2_ fragment conjugated to Alexa Fluor 488 fluorescent dye and anti-rabbit IgG (H + L), F(ab’)_2_ fragment conjugated to Alexa Fluor 555 fluorescent dye (#4408 & #4413, Cell Signaling Technology, Germany; 1:1000 in blocking solution) for 45 min at room temperature. Following 2x 5 min in 0.015% Triton X-100 in PBS and 2x 10 min in PBS, slides were mounted with 16 µL Vectashield mounting medium including 4′-6-diamidino-2-phenylindole (DAPI) (Vector Laboratories, USA). Immunofluorescence analysis and image acquisition were performed using the epifluorescence microscope Zeiss AxioImager.Z2 (objectives: ECPlnN 20x/0.50 M27; ECPlnN 40x/0.75 M27; EC-PlnN 63x/0.95 Korr M27and filters: Zeiss 96 HE BFP, Zeiss 38, Zeiss 43) equipped with Axiocam 503 mono, LED light source Colibri7 (Carl Zeiss, Germany) and Zen Software (Carl Zeiss, Germany). The number of radiation-induced γH2AX, 53BP1 and colocalized foci were manually quantified in 100 cells (when available) on recorded pictures. Data analysis was carried out by using a generalized linear model with a quasipoisson error distribution.

### Radiation-impaired cellular function

Radiation-impaired cellular function is demonstrated by reduced cellular migration and proliferation, which was called a spreading assay. Keratinocytes were harvested as described above, cell suspension was adjusted to a density of 20,000 cells/mL and divided into 500 µL aliquots in 1.5 mL polypropylene tubes. Tubes with cell suspension were then placed upright in a fixed position within the irradiation chamber of a ^137^Cs Gammacell 40 Exactor (Best Theratronics, Canada) and exposed to 0, 2, 4, 6 or 8 Gy at a dose rate of 0.63 Gy min^−1^. Irradiation was performed at room temperature and in adherence to the manufacturer’s instructions. Additional information on the irradiation setup is given in Suppl. Figure 7.

Cells were seeded in triplicates at 3000 cells (150 µL) into polydimethylsiloxane (PDMS) rings within 12-well-plates; PDMS rings were manufactured as illustrated in Suppl. Figure 2, autoclaved and dried under a sterile hood. Assay setup is displayed in Suppl. Figure 1. After 6–8 h of cellular growth, PDMS rings were removed, and each well was flooded with 2 mL of the appropriate culture medium.

Cells were allowed to proliferate and expand for 11 days. Thereafter medium was replaced with 0.05% crystal violet (w/v), 1% formaldehyde, and 1% methanol in 1X PBS [9] for 20 min at room temperature; fixed and stained cells were washed three times with demineralized water and dried. To provide a homogeneous background for scanning plates, we placed custom-made white polyether ether ketone (PEEK) inserts on top of the fixed cells (Suppl. Figure 3). Scans of the cell-covered areas were taken from the bottom of the plates at 600 dpi with a high-resolution flatbed scanner (CanoScan 9000F Mark II, Canon Inc.) using the reflected light modus, and stored as TIF files. Area was quantified using ImageJ image analysis software (NIH) as shown in Suppl. Figure 6.

To validate the planimetric measurements above, we quantified the amount of crystal violet bound to the cells. Dye was dissolved in 500 µL of 70% ethanol per well and optical density was measured at 590 nm in a microplate spectrophotometer.

## Colony forming efficiency of oral keratinocytes using hMSC feeder layers

To confirm the radiation-impaired cellular function, the colony forming efficiency (CFE) of keratinocytes grown in coculture with hMSC was tested (Suppl. Figure 5). Passage 2–4 hMSC were seeded at 5000 cells/cm^2^ into 6-well plates in MSC medium (DMEM/F12 (Invitrogen) containing 5% autologous human serum and supplements [6]. Cells were allowed to adhere for 24 h and overlaid thereafter with keratinocytes at 10 cells/cm^2^; keratinocyte cell density was adjusted according to the radiation dose: 20 cells/cm^2^ at 2 Gy, 60 cells/cm^2^ at 4 Gy, 120 cells/cm^2^ at 6 Gy, and 240 cells/cm^2^ at 8 Gy. On day 9, keratinocyte colonies (>50 cells) were stained with crystal violet and counted. CFE was calculated by dividing the number of counted colonies by the number of keratinocytes seeded.

### Bioinformatical analysis of survival and migration data

To investigate the impact of cell-covered area (spreading assay), radiation dose and group (healthy volunteer or patient) on the colony formation efficiency (CFE), we applied a linear mixed model with log(CFE) as dependent variable and area, radiation dose and group as covariates. Log CFE was modelled depending on the radiation dose with the individual as a random factor. In a second model, log CFE was modelled dependent on both the radiation dose and spreading assay. We used the open-source statistical software environment R (version 4.1.0) and the R package lme4 (version 4_1.1–21) [10], [11]. Details on statistical analyses are provided in supplementary files 3 and 4.

Individual subjects’ radiation-impaired cell spread was additionally estimated by principal component analysis (PCA): spread areas at 0, 2, 4, 6 and 8 Gy were scaled at the particular dose level and first component PCA values were normalized by the square root of the eigen value to allow comparison between individuals. Analyses were performed using JMP 8.0 statistical discovery software.

## Results

### Keratinocytes grow out from mucosa micro-biopsies

**Fig. 1:**
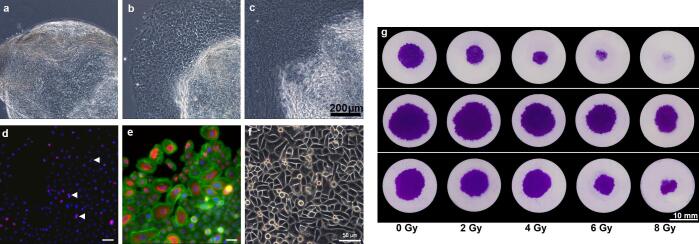
Kinetics, properties and spreading assays of explant human oral keratinocytes. Outgrowth of keratinocytes from an oral mucosa particle on (a) day 0, (b) day 1 and (c) day 2, phase contrast microscopy. (d) – (f) Properties of primary human oral keratinocytes: (d) Immunofluorescence for Ki-67 (pink) shows proliferating cells; arrowheads indicate mitotic figures; nuclei (DAPI, blue). (e) Immunofluorescence for cytokeratin 5/14 (red), cytoskeleton (phalloidin) (green) and nuclei (DAPI, blue). (f) Following subcultivation, keratinocytes continued to grow as a monolayer of polygonal cells, phase contrast microscopy. (g) Spreading assay from primary keratinocytes, 3 individuals with high (upper row), low (middle row) and intermediate (lower row) radiosensitivity, following 0, 2, 4, 6 and 8 Gy exposure. (For interpretation of the references to colour in this figure legend, the reader is referred to the web version of this article.)

One week after biopsy complete mucosa healing was documented in all subjects. One hundred and forty-four gingiva particles from 16 individuals were processed by micro-dissection (age: 29 to 65 years; median 47) resulting in 111 primary keratinocyte cultures (success rate: 85%). Cells attached to the culture dish within 24 to 48 h and started exuding out of the tissue particle (Fig. 1). Substantial numbers of mitotic cells occurred at day 2 or 3 (Fig. 1d). Immunofluorescence for cytokeratin 5/14 confirmed the keratinocyte identity [7] (Fig. 1e). After passage, cells maintained a polygonal morphology, forming densely packed monolayers (Fig. 1f). Expansion of keratinocytes was successful 7 out of 8 HNSCC patients, and in all 8 healthy individuals.

Continuous proliferation (three passages, mean; range: 2–6) for 4–7 weeks was documented for 14 out of 15 individuals (93%). The average doubling time in the first passage was 55 h (range: 33–105). Thereafter, proliferation rates declined, and cells changed to a more flattened, less compact morphology. Upon recultivation following liquid nitrogen preservation, cells from 12 out of 15 individuals showed normal growth.

### Radiation-induced double strand breaks in oral keratinocytes increases after 24 and 96 h of repair

We used residual DNA damage in relation to non-irradiated cells as a measure for the DNA repair capacity. Upon exposure to 6 Gy, after 24 h of repair, residual colocalized nuclear gammaH2AX/53 BP1 foci were increased 3.7-fold (mean) compared to non-irradiated control samples. At 96 h repair time, the irradiated samples displayed less residual damage than at 24 h, but still foci numbers were slightly increased by 0.6-fold (mean) compared to the non-irradiated control for most of the individuals. For some individuals, negative values result from fewer foci in the irradiated cells compared to non-irradiated cells. Therefore, except for P30 (Table 1), DNA damage was almost completely repaired to control levels. Raw data is shown in Suppl. Table 2.

**Table 1:** Residual colocalized gammaH2AX/ 53 BP1 foci after 6 Gy, corrected for the respective control at 0 Gy by subtracting the respective means; (mean; confidence interval).

Individual	Radiation-associated increase in focus numbers	
	**24 h post radiation**	**96 h post radiation**
**P52**	**9.41** (6.05 – 13.51)	**−0.37** (-0.84 – 0.07)
**P53**	**2.42** (1.35 – 3.74)	**0.23** (-0.72 – 1.09)
**P51**	**1.61** (0.68 – 2.66)	**0.74** (0.33 – 1.16)
**P31**	**2.92** (2.22 – 3.69)	**0.89** (0.33 – 1.50)
**P49**	**3.52** (2.71 – 4.41)	**−0.38** (-1.49 – 0.72)
**P30**	**2.87** (1.91 – 3.92)	**1.87** (0.92 – 2.88)

### Cellular spreading of established cell lines and oral keratinocytes correlates to radiation dose

PDMS rings confine attachment of Cal27, Cal33 and OKF6 cells to an area of 16.6 mm^2^ for 6 to 8 h. Following removal of the PDMS rings and after 10 days of cultivation, OKF6-cells expanded to 68.5 mm^2^ (median; range: 41.6–74.4). Radiation delayed expansion in a dose dependent manner (Fig. 1G and H). Comparable attachment and dose-dependent expansion was observed for Cal27 and Cal33 cells.

**Fig. 2:**
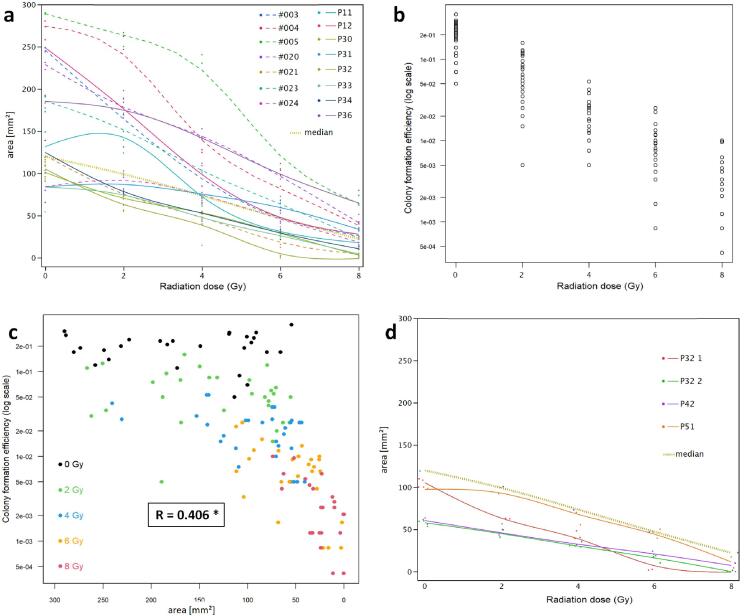
Results of radiation resistance testing of biopsy-derived oral keratinocytes from individual subjects. (a) Cellular spread following 0, 2, 4, 6 and 8 Gy exposure; 15 individuals, colour-coded triplicates and appropriately fitted lines, and median of all subjects (dotted line); P = healthy individual; # = patient. (b) Colony formation efficiency of oral keratinocytes following 0, 2, 4, 6 and 8 Gy exposure; 15 individuals. (c) Cell spread correlates to clonal growth of oral keratinocytes. Radiation doses are coded by colour. *Pearson correlation coefficient. (d) Cellular spread of keratinocytes from a single healthy individual following 0, 2, 4, 6 and 8 Gy exposure; colour-coded triplicates (if applicable) and appropriately fitted lines; P32, P42 and P51 = different biopsies or cell harvest (P32_1 = passage 1; P32_2 = passage 2). Note: all observations are below the median of all 15 subjects tested (dotted line), which indicates that keratinocytes from this individual have a consistently high radiosensitivity.

PDMS rings confined attachment of oral keratinocytes to 16.6 mm^2^ for 6 to 8 h. Following removal of rings cells expanded within 10 days to 119.2 mm^2^ (median; range: 54.4–290). Radiation before seeding reduced spreading of the keratinocytes to 100.7 mm^2^ (median; range: 55.3–266.7) at 2 Gy; 73.2 mm^2^ (median; range: 15–240.4) at 4 Gy; 47 mm^2^ (median; range: 2–111.9) at 6 Gy, and 22.7 mm^2^ (median; range: 0–80) at 8 Gy (Suppl. Table 1). The overall linear slope from 0 to 8 Gy amounts to −16.95. Differences in resistance to radiation between individuals (Fig. 2) may be best discriminated visually between 4 and 6 Gy because these slopes show the best parallel alignment (Fig. 2a). Planimetric measurements of the cell-covered area correlated to cellular bound crystal violet, and related to the radiation dose applied (p < 0.0001; Suppl. Figure 6).

To more conveniently compare cell spread of samples from different individuals (Fig. 2a) and from one individual at different biopsy dates or different cell harvest passages (Fig. 2d), the dose dependent cellular spread was parametrized by a principal component analysis (PCA); variance of the first component is 88.4; relevant data are depicted in Table 2 and allow one to rank individuals for resistance to radiation in a numerical order. PCA confirms the interpretation of the visual graphic presentation in all cell samples and correlates with the overall linear slope (p < 0.0001; Table 2).

**Table 2:** Normalized first component values (PC_1) of cellular spreading at 0, 2, 4 and 6 Gy exposure and overall linear slope (calculated from 0 to 8 Gy); 15 individuals; P = healthy individual; # = patient; * = cells from one single individual at different biopsy dates (P32, P42 and P51) or a different cell passage (P32_1; P32_2).

**Individual**	**PC_1**	**slope**
P11	−0.206	−16.9
P12	0.543	−28.5
P30	−0.768	−11.9
P31	−0.143	−6.7
P32_1*	−1.045	−13.5
P33	−0.831	−10.3
P34	−0.598	−13.8
P36	1.277	−15.6
#003	0.450	−27.8
#004	1.322	−31.5
#005	2.315	−29.6
#020	1.169	−22.8
#021	−0.779	−14.5
#023	0.369	−20.6
#024	−0.381	−8.9
P32_2*	−1.180	−7.2
P42*	−1.055	−6.6
P51*	−0.457	−10.7
Median	−0.294	−14.2
Range	−1.18 to 2.315	−31.5 to −6.6

When seeded onto non-irradiated bmMSC feeder layers, the median colony formation efficiency (CFE) of oral keratinocytes was 0.223 in non-irradiated controls, range: 0.05–0.32, i.e., around 20% of the seeded keratinocytes gave rise to colonies of > 50 cells. After radiation, CFE decreased in a dose dependent manner, to 0.0028 (median; following 8 Gy; Fig. 2b). Fig. 2c depicts the individual results.

A mixed model with individual donors as a random factor revealed that the delay of cell spread correlated with decreased CFE (Fig. 2c); the main driver is radiation dose (decrease by a factor of 0.50, adjusted for area). Spread area itself had no direct effect.

The log-transformed CFE decreased by 0.683 per 1 Gy, which means shrinking by a factor of about 50% per 1 Gy for the untransformed CFE. Area and group showed no additional impact on the CFE. See supplemental file 3 for R code and results.

If individuals undergo subsequent gingival biopsies, a dose-dependent delay of the cell spread is reproducibly observed. Although repeated tests can not be exactly superimposed graphically, the overall slopes and PC_1 values suggest that this individual’s cells are always more radiosensitive than the median of all individuals tested (Fig. 2d and Table 2).

## Discussion

Severe side effects following radiotherapy of head and neck cancer occur frequently and show substantial inter-individual variation [12]. The regenerative capacity of oral mucosa following ionizing radiation is not fully understood [13]. Radiation responses have been looked at in mucosa smears [14], biopsies [1], [15], [16], explants [17] and reconstituted, three-dimensional tissue models [18], [19], [20], [21], [22], but most reports focus on the cellular function of keratinocytes [7], [12]. However, assays of keratinocytes to estimate an individual subject’s response to radiation are not available.

Here, we report for the first time on an oral mucosa micro-biopsy technique performed on individually retrieved oral keratinocytes that is designed to predict radiation toxicity for patients with cancer of the head and neck area. This technique causes minimal discomfort, is safe and allows lesions to completely recover in healthy individuals and patients within one week. Although our tissue samples are smaller than previously reported [23], we achieve reliable cellular growth. Outgrowing cells display keratinocyte characteristics and respond to radiation with typical DNA-damage. Keratinocytes may easily be cryopreserved and remain available for further functional assays such as a gammaH2AX/ 53BP1 foci assay potentially predicting radioresistance [24].

Clonogenic tests for radioresistance, although readily available for cell lines [25], are difficult to perform with biopsy-derived keratinocytes because these have low colony formation efficacies when seeded without feeder cells [18], [26], [27]. Here we describe a feeder cell-free assay that investigates how radiation affects proliferation and migration of keratinocytes, both features involved in, and essential for filling “empty space” [28], [29] resulting from damaged and denuded mucosa. The observed cell spread correlates to the radiation dose applied and is consistent for the cells of all subjects tested. Diagrammed results (Fig. 2) and principal component analyses (Table 2) allowed us to rank individual donors according to their radioresistance. Our findings were, further, validated by colony formation efficiency testing, which had an intermediate correlation coefficient between the two assay types.

Of note: Although repeated tests from single subject’s cells provided here an assessment for radioresistance, they do express some heterogeneity probably because of short-lived and heterogenous primary oral keratinocytes. A small sample size limits our data. Finally, the clinical relevance still has to be proven.

We conclude that robust *in vitro* growth of keratinocytes from clinical micro-biopsies is feasible. Our test for migration and proliferation capacities allows one to estimate the radioresistance. Cells may be used for personalized models to investigate radiation-induced damage and additional cellular features [30], [31]. Studies on the clinical relevance are presently underway, which include a larger sample size and detailed evaluation of mucositis.

## Declaration of Competing Interest

The authors declare that they have no known competing financial interests or personal relationships that could have appeared to influence the work reported in this paper.

## References

[1] Dörr W., Hamilton C.S., Boyd T., Reed B., Denham J.W. (2002). Radiation-induced changes in cellularity and proliferation in human oral mucosa. Int J Rad Oncol Biol Phys.

[2] Maria O.M., Eliopoulos N., Muanza T. (2017). Radiation-Induced Oral Mucositis. Front. Oncol.

[3] Gomolka M., Blyth B., Bourguignon M., Badie C., Schmitz A., Talbot C. (2020). Potential screening assays for individual radiation sensitivity and susceptibility and their current validation state. Int J Radiat Biol.

[4] Elhalawani H., Cardenas C.E., Volpe S., Barua S., Stieb S., Rock C.B. (2021). 18FDG positron emission tomography mining for metabolic imaging biomarkers of radiation-induced xerostomia in patients with oropharyngeal cancer. Clin Transl Radiat Oncol.

[5] Ophof R., van Rheden R.E.M., von den Hoff J.W., Schalkwijk J., Kuijpers-Jagtman A.M. (2002). Oral keratinocytes cultured on dermal matrices form a mucosa-like tissue. Biomaterials.

[6] Thomsen A.R., Aldrian C., Bronsert P., Thomann Y., Nanko N., Melin N. (2017). A deep conical agarose microwell array for adhesion independent three-dimensional cell culture and dynamic volume measurement. Lab Chip.

[7] Bucchieri F., Fucarino A., Marino Gammazza A., Pitruzzella A., Marciano V., Paderni C. (2012). Medium-term culture of normal human oral mucosa: a novel three-dimensional model to study the effectiveness of drugs administration. Curr Pharm Des.

[8] Bucher M., Duchrow L., Endesfelder D., Roessler U., Gomolka M. (2020). Comparison of inexperienced operators and experts in γH2A.X and 53BP1 foci assay for high-throughput biodosimetry approaches in a mass casualty incident. Int J Radiat Biol.

[9] Feoktistova M, Geserick P, Leverkus M. Crystal Violet Assay for Determining Viability of Cultured Cells. Cold Spring Harb Protoc 2016;2016(4):pdb.prot087379. 10.1101/pdb.prot087379. 10.1101/pdb.prot087379 27037069

[10] Bates D, Mächler M, Bolker B, Walker S. Fitting Linear Mixed-Effects Models Using lme4. J. Stat. Soft. 2015;67(1). 10.18637/jss.v067.i01.

[11] R Core Team (2021).

[12] Fowler J.F., Harari P.M., Leborgne F., Leborgne J.H. (2003). Acute radiation reactions in oral and pharyngeal mucosa: tolerable levels in altered fractionation schedules. Radiother Oncol.

[13] Jones K.B., Klein O.D. (2013). Oral epithelial stem cells in tissue maintenance and disease: the first steps in a long journey. Int J Oral Sci.

[14] Patil V., Baad R., Gudur A., Vibhute N., Belgaumi U., Kadashetti V. (2018). Evaluation of Radiation-induced Cytological Changes in Lesional Oral Cancer Cells and Adjacent Normal Mucosal Cells. J Contemp Dent Pract.

[15] Marcussen M., Sønderkær M., Bødker J.S., Andersen M., Nielsen S., Vesteghem C. (2018). Oral mucosa tissue gene expression profiling before, during, and after radiation therapy for tonsil squamous cell carcinoma. PLoS ONE.

[16] Handschel J., Prott F.-J., Sunderkötter C., Metze D., Meyer U., Joos U. (1999). Irradiation induces increase of adhesion molecules and accumulation of β2-integrin-expressing cells in humans. International Journal of Radiation Oncology*Biology*Physics.

[17] Donetti E., Bedoni M., Capone P., Gualerzi A., Tartaglia G., Sforza C. (2009). An in vitro model of human oral explants to study early effects of radiation mucositis. Eur J Oral Sci.

[18] Tobita T., Izumi K., Feinberg S.E. (2010). Development of an in vitro model for radiation-induced effects on oral keratinocytes. Int J Oral Maxillofac Surg.

[19] Rakhorst H.A., Tra W.M.W., Posthumus-Van Sluijs S.T., Hovius S.E.R., Levendag P.C., Kanaar R. (2006). Quantitative analysis of radiation-induced DNA break repair in a cultured oral mucosal model. Tissue Eng.

[20] Colley H.E., Eves P.C., Pinnock A., Thornhill M.H., Murdoch C. (2013). Tissue-engineered oral mucosa to study radiotherapy-induced oral mucositis. Int J Radiat Biol.

[21] Lambros M.P., DeSalvo M.K., Mulamalla H.C., Moreno J., Kondapalli L. (2016). Genome wide expression after different doses of irradiation of a three-dimensional (3D) model of oral mucosal. Genom Data.

[22] Tschachojan V., Schroer H., Averbeck N., Mueller-Klieser W. (2014). Carbon ions and X-rays induce pro-inflammatory effects in 3D oral mucosa models with and without PBMCs. Oncol Rep.

[23] Lauer G., Mai R., Pradel W., Proff P., Gedrange T., Beyer J. (2006). Influence of Cyclosporin A on human gingival keratinocytes in vitro. Journal of Cranio-Maxillofacial Surgery.

[24] Subedi P., Gomolka M., Moertl S., Dietz A. (2021). Ionizing Radiation Protein Biomarkers in Normal Tissue and Their Correlation to Radiosensitivity. A Systematic Review J Pers Med.

[25] Puck T.T., Marcus P.I. (1955). A rapid method for viable cell titration and clon production with HeLa cells in tissue culture: The use of X-irradiated cells to supply conditioning factors. Proc Natl Acad Sci U S A.

[26] Cheshire P., Zhafira A.S., Banakh I., Rahman M.M., Carmichael I., Herson M. (2019). Xeno-free expansion of adult keratinocytes for clinical application: the use of human-derived feeder cells and serum. Cell Tissue Res.

[27] Slonina D., Hoinkis C., Dörr W. (2001). Effect of keratinocyte growth factor on radiation survival and colony size of human epidermal keratinocytes in vitro. Radiat Res.

[28] Blanpain C., Horsley V., Fuchs E. (2007). Epithelial stem cells: turning over new leaves. Cell.

[29] Hoshikawa E, Sato T, Kimori Y, Suzuki A, Haga K, Kato H et al. Noninvasive measurement of cell/colony motion using image analysis methods to evaluate the proliferative capacity of oral keratinocytes as a tool for quality control in regenerative medicine. J Tissue Eng 2019;10:2041731419881528. 10.1177/2041731419881528. 10.1177/2041731419881528 PMC6794654 31662840

[30] Chmara J., Browning J.W.L., Atkins H., Sabloff M., McKay B.C. (2018). Rapid Decrease in KRT14 and TP53 mRNA Expression in the Buccal Mucosa of Patients Receiving Total-Body Irradiation for Allogeneic Stem Cell Transplantation. Radiat Res.

[31] Degen M., Wiederkehr A., La Scala G.C., Carmann C., Schnyder I., Katsaros C. (2018). Keratinocytes Isolated From Individual Cleft Lip/Palate Patients Display Variations in Their Differentiation Potential in vitro. Front Physiol.

