# Supplementary material for: Biopsy-derived oral keratinocytes – A model to potentially test for oral mucosa radiation sensitivity
Source: Clin Transl Radiat Oncol. 2022 Mar 16;34:51–6. doi: 10.1016/j.ctro.2022.03.007 (PMC8956846; doi:10.1016/j.ctro.2022.03.007)
Supplement: Supplementary data 1 [file mmc1.docx]

**Supplemental file 1 to**

**Biopsy-Derived Oral Keratinocytes – a Model to Potentially Test for Oral Mucosa Radiation Sensitivity**

**by Thomsen AR, Aldrian C et al.**


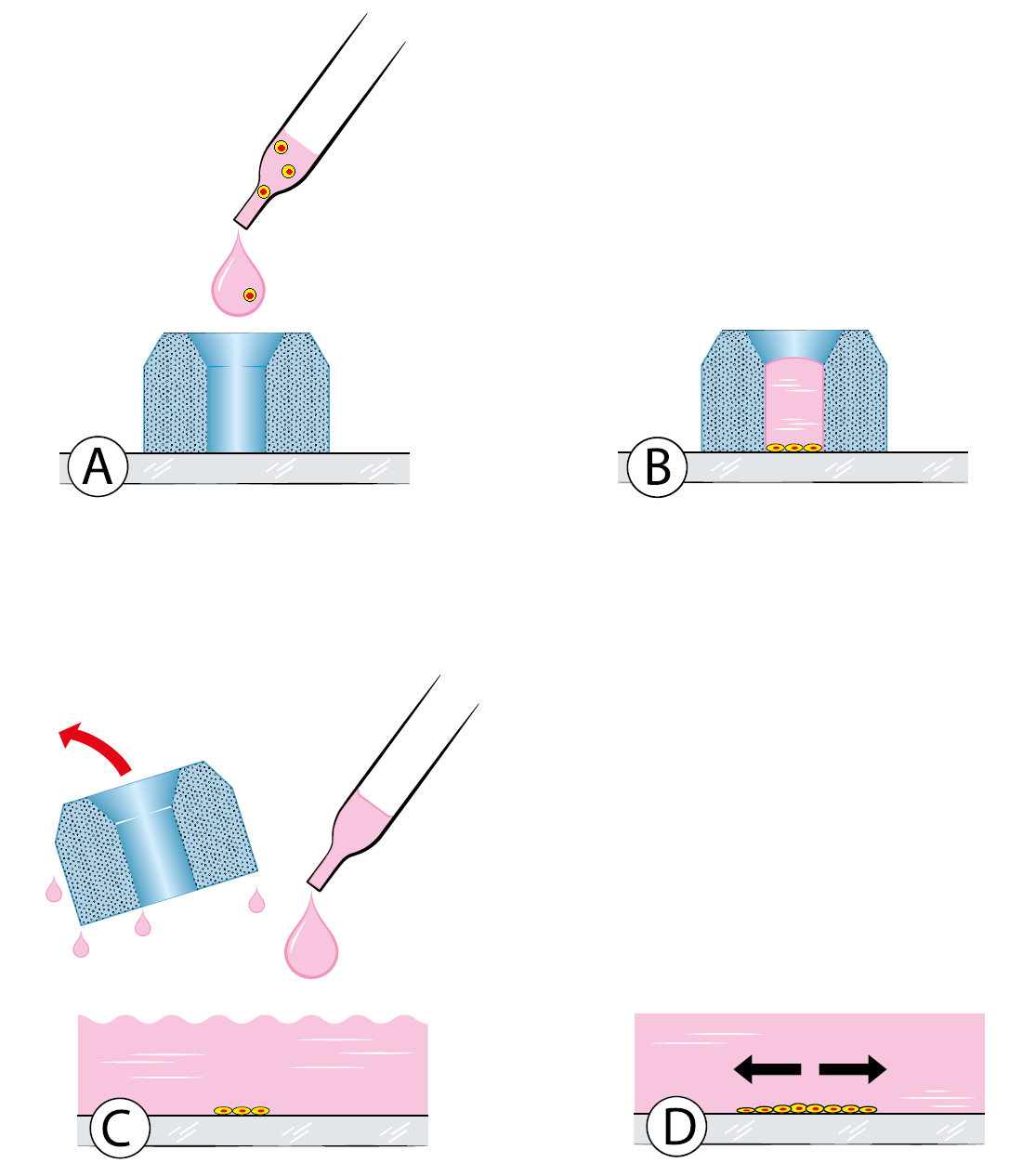


**Suppl. Figure 1: Setup of spreading assay. (A) The cell suspension is filled into the silicone ring. (B) Cells sediment and attach to the culture surface. (C) 6-8 hours later, the silicone ring is removed, and the well is flooded with culture medium. (D) The resulting cell cluster spreads over a period of 11 days.**

**
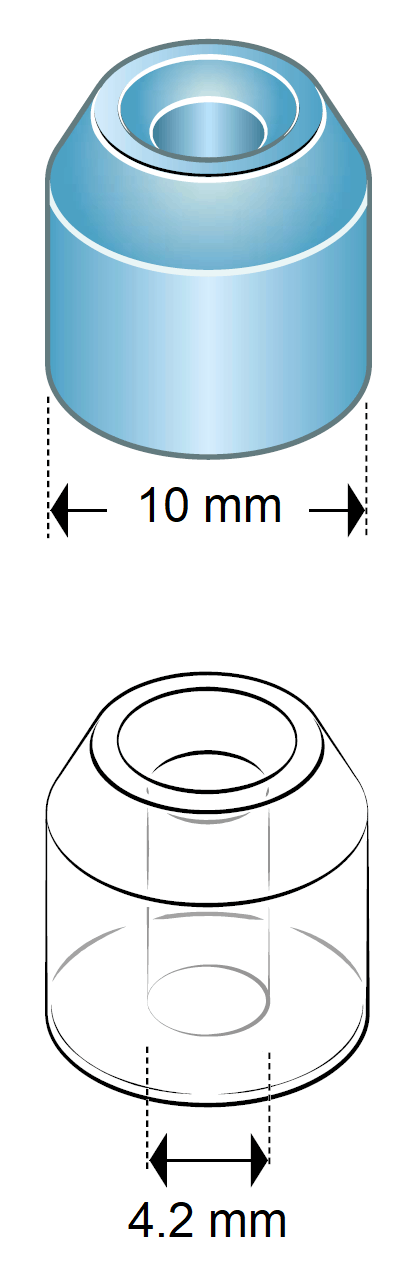

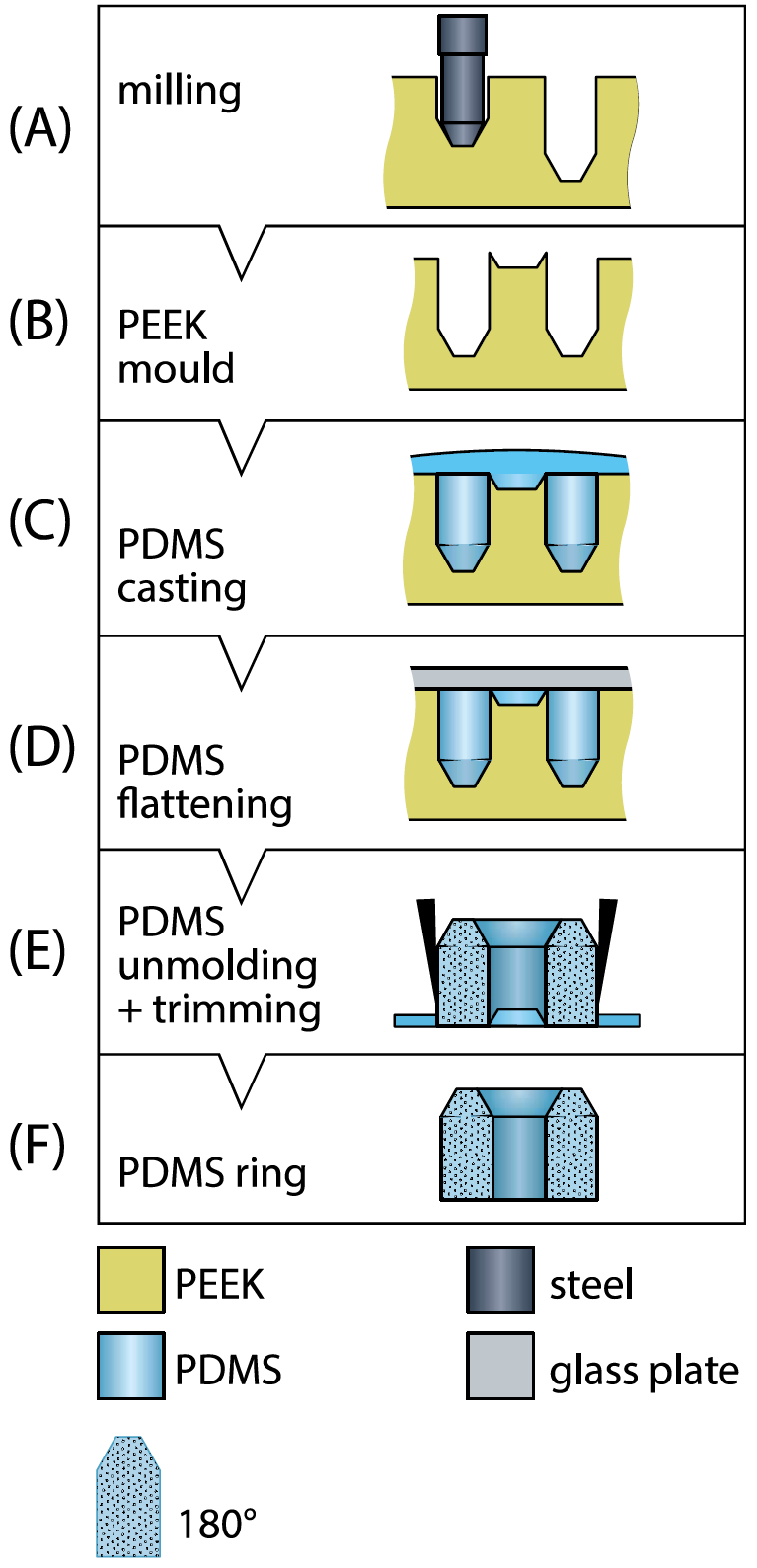
**

(G)

(H)
 **
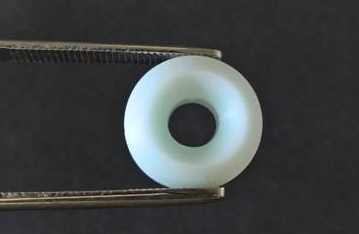
**

**Suppl. Figure 2: Manufacturing of PDMS rings for the spreading assay. The geometry is defined by computer-aided design. (A) Negative shapes are then machined into polyether ether ketone (PEEK) under computerized numerical control (CNC), using a VCP 800 3-Axis CNC mill (Mikron, Germany). (B, C) The resulting PEEK moulds are cast with PDMS, followed by centrifugation (1 min, 1200 × g) to remove bubbles. (D) Glass plates are placed on the PDMS to create a smooth surface. (E, F) After polymerization, the PDMS rings are unmoulded and trimmed to obtain the final rings, which are boiled in demineralized water for 30 min to remove unbound monomers, and then autoclaved in water for 15 min at 121°C. (G) Dimensions of a PDMS ring. (H) Photograph of a PDMS ring being held by forceps.**


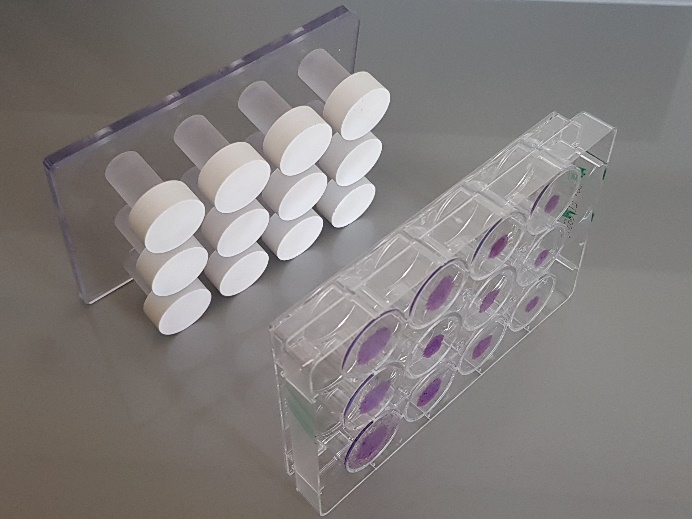

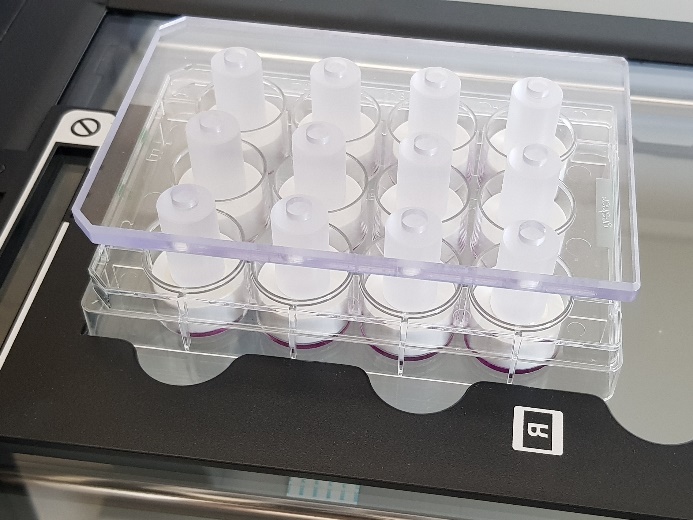


**Suppl. Figure 3: Scanning set-up using PEEK inserts**.

**
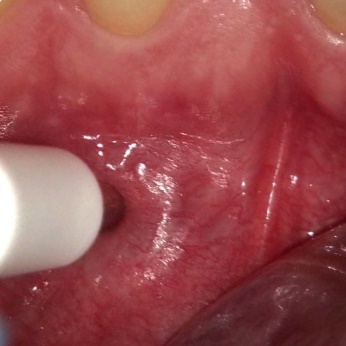
**
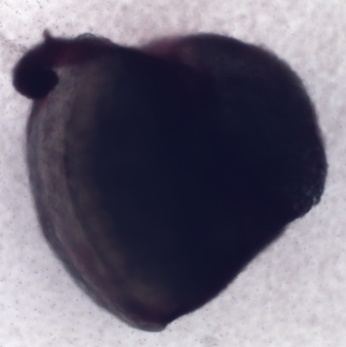

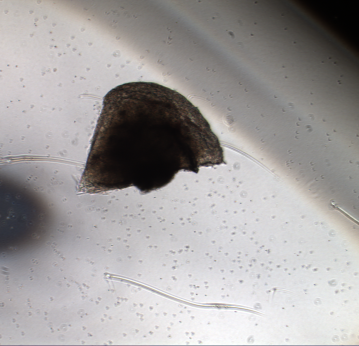


**500 µm**

**500 µm**

(A) (B) (C)

**Suppl. Figure 4: Oral mucosa harvesting and dissection. (A) View of the biopsy punching process. Microscopy of oral mucosa tissue after biopsy: (B) Whole punch, diameter 1.54 mm, and (C) one out of 6 segments cut out of the tissue.**


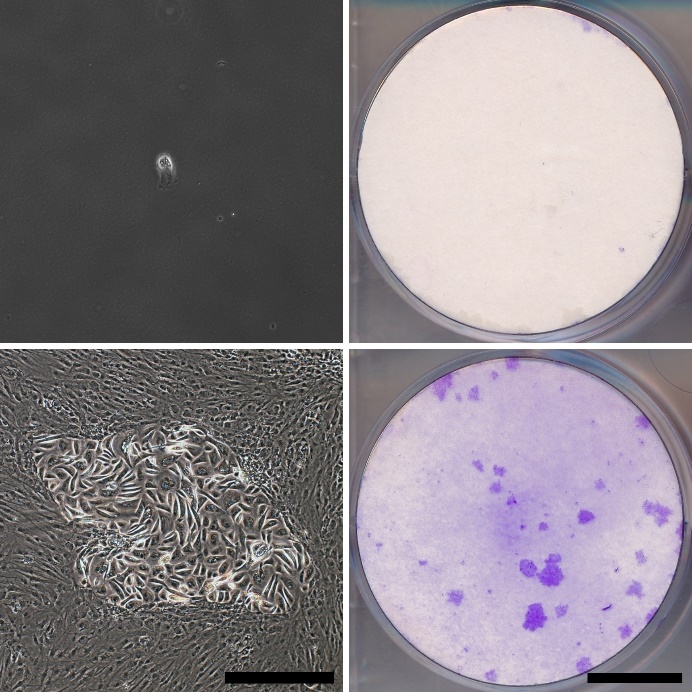


Oral keratinocytes alone

Oral keratinocytes
+ bmMSC

**(A)**

**(B)**

**200 µm**

**10 mm**

**Suppl. Figure 5: Colony formation of primary human oral keratinocytes in the absence and presence of bMSC feeders. On day 9 after seeding 100 oral keratinocytes per well in a 6-well plate, colonies have only formed in the presence of bmMSC feeders. (A) Phase contrast microscopy, and (B) scan of complete wells after staining with crystal violet.**


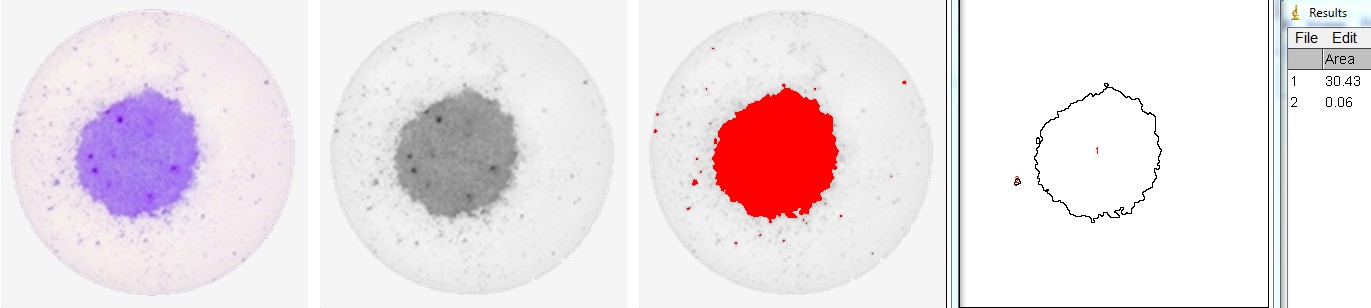


**Suppl. Figure 6: Measurement of the cell spreading area, following the spreading assay.
(A) Scan of stained cell cluster, original resolution 600 dpi. (B) Scan converted into a greyscale image. (C) Threshold applied in ImageJ image analysis software (NIH) (D) Resulting contours and area values.**


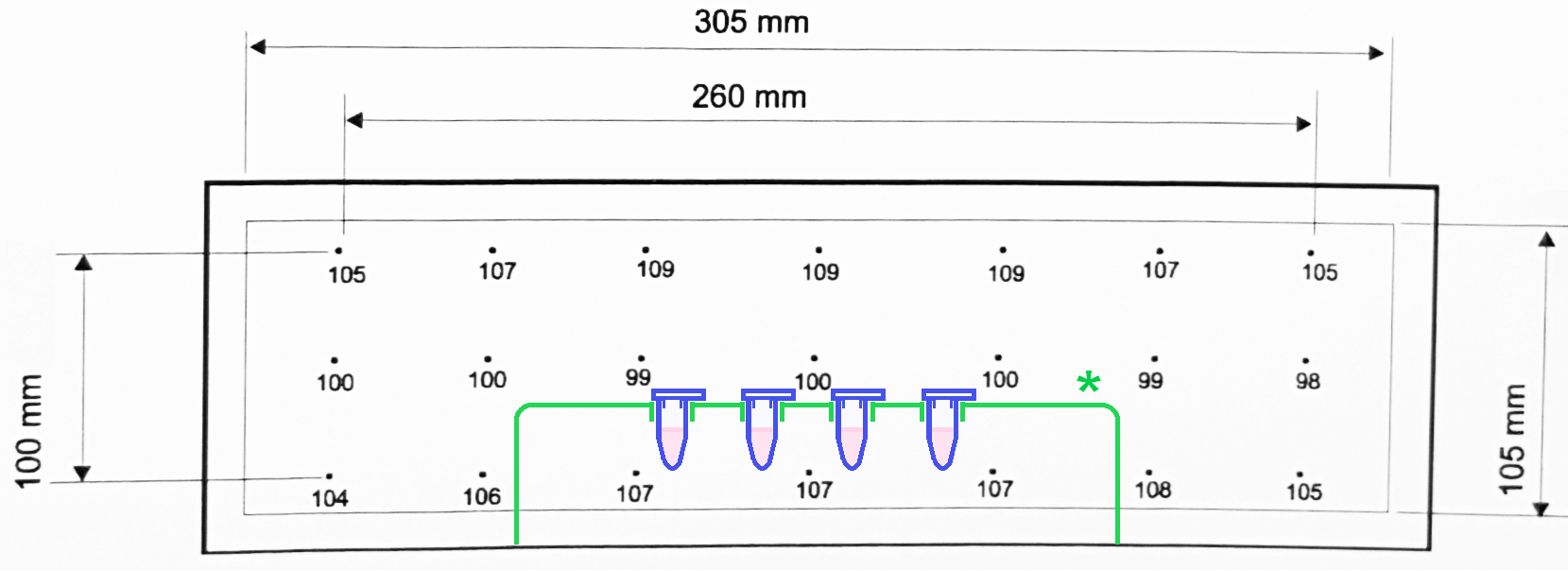


**Suppl. Figure 7: Relative distribution of absorbed dose (% of set value) within the irradiation chamber of the ^137^Cs Gammacell 40 Exactor (Best Theratronics, Canada) as provided by the manufacturer, overlayed with a scheme of the rack (*) and tubes containing keratinocyte suspension.
Accuracy of dose distribution inside the irradiation chamber had previously been checked using TLD by the physics department of the Freiburg radiation oncology.**

**(A)**

**(B)**

**Suppl. Figure 8: Planimetric (A) versus colorimetric (B) evaluation of spreading assays. Results from three different individuals (D1-D3). These keratinocyte cultures (from three healthy donors) originate from pre-tests and therefore are not part of the study cohort.**


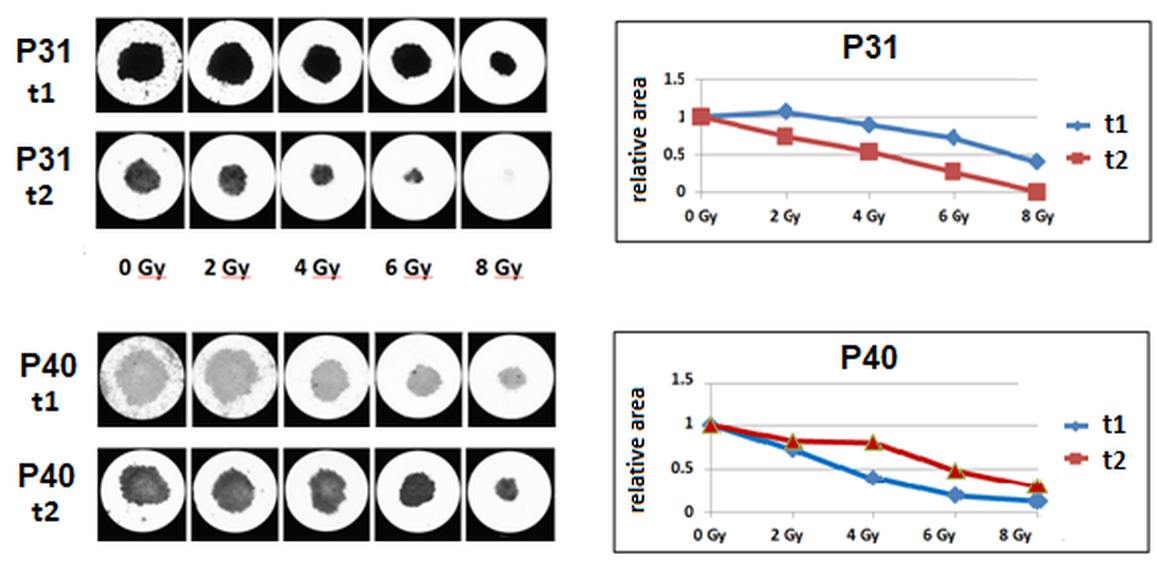


**Suppl. Figure 9: Spreading assays performed after a first (t1) and a second biopsy (t2, 6 months later) from two individual persons. Left: representative scans of cell spreading areas. Right: mean area of cell spreading areas, normalized to 0 Gy. P31 t2, P40 t1 and P40 t2 represent samples from healthy donors which were not included into the study cohort, because the respective cell cultures were performed at a later stage.**

**
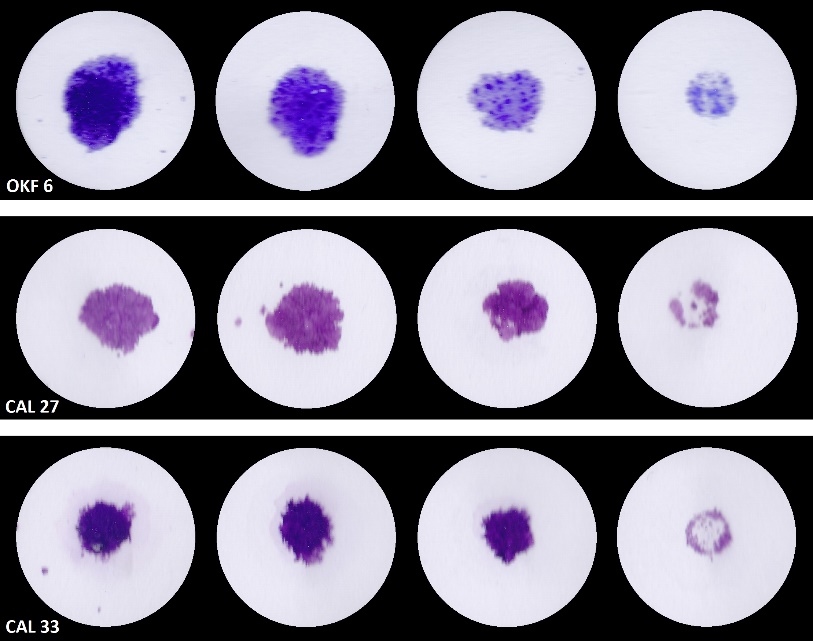
**

**Suppl. Figure 10: Spreading assay from a normal mucosa-derived cell line (OKF 6) and 2 head and neck cancer cell lines (Cal 27 and Cal 33) following 0, 2, 4 and 6 Gy exposure.**

**Suppl. Table 1: Cellular spreading area (mm^2^) following 0, 2, 4 and 6 Gy exposure; triplicates (if available); 15 individuals; P = healthy individual; # = patient; *=same individual at different biopsy dates (P32, P42 and P51) or from one biopsy and cell harvest at a different cell passage (P32-1; P32-2)**

| **Individual** | **0 Gy** | **2 Gy** | **4 Gy** | **6 Gy** | **8Gy** | |
| --- | --- | --- | --- | --- | --- | --- |
| #003 | 243.9 | 131.4 | 74.5 | 43.6 | 32.5 |  |
| #003 | 248.9 | 198.2 | 112.3 | 47 | 23.2 |  |
| #004 | 280.4 | 262.3 | 124.9 | 68.3 | 35.2 |  |
| #004 | 258.1 | 246.8 | 128.1 | 103.9 | 41.4 |  |
| #005 | 288.5 | 266.7 | 240.4 | 105.5 | 73.9 |  |
| #005 | 290 | 250.5 | 230.5 | 111.7 | 62.7 |  |
| #020 | 231.3 | 165.5 | 153 | 98.3 | 40.2 |  |
| #020 | . | 184.3 | 141.6 | 93.5 | 42.9 |  |
| #020 | . | 175.1 | 135 | 103.9 | 45.3 |  |
| #021 | 113.6 | 73.7 | 54.7 | 21.6 | 2.7 |  |
| #021 | 149 | 69.7 | 51.9 | 15.9 | 11.4 |  |
| #021 | 103.7 | 76.4 | 45.6 | 12.5 | 1.9 |  |
| #023 | 173.1 | 139.6 | 99.2 | 65 | 23.9 |  |
| #023 | 190.7 | 149.6 | 101.8 | 56.2 | 23.4 |  |
| #023 | 191.3 | 171.3 | 103.7 | 72.9 | 22.2 |  |
| #024 | 80.1 | 95.6 | 70.2 | 47.7 | 23.8 |  |
| #024 | 80 | 81.9 | 67.9 | 47.6 | 18.2 |  |
| #024 | 90.7 | 103.1 | 85.4 | 41.3 | 14.4 |  |
| P11 | 149 | 188.2 | 54.4 | 33 | 21.9 |  |
| P11 | 103.5 | 124.4 | 70.7 | 31.7 | 15.9 |  |
| P12 | 222.9 | 189.1 | 84.7 | 56.5 | 33.7 |  |
| P12 | 273.5 | 169.1 | 109.3 | 36.6 | 25.6 |  |
| P30 | 96.2 | 72.9 | 55.3 | 28.1 | 0.5 |  |
| P30 | 93.4 | 55.3 | 46.3 | 25.6 | 3.8 |  |
| P30 | 116.3 | 79 | 63.4 | 32.2 | 5.6 |  |
| P31 | 100.8 | 98.2 | 72 | 53.2 | 31.9 |  |
| P31 | 65.8 | 79.5 | 74.3 | 68 | 35.8 |  |
| P31 | . | . | 77.6 | 60.2 | 32.9 |  |
| P32-1 | 100.2 | 63.3 | 40.6 | 3 | 0.2 |  |
| P32-1 | 108.5 | 56.7 | 48.4 | 2 | 1.6 |  |
| P32-1 | 110 | 62.5 | 39.3 | . | 0.1 |  |
| *P32-2 (kryo) | 60.3 | 50.07 | 29.25 | 10.5 | 0.2 |  |
| *P32-2 (kryo) | 53.7 | 41.13 | 30.33 | 20.4 | 0.13 |  |
| *P32-2 (kryo) | 59.7 | 44.71 | 30.79 | 19.1 | 0 |  |
| P33 | 91.2 | 78.1 | 61 | 24.7 | 0 |  |
| P33 | 54.4 | 70.7 | 62.4 | 25.6 | 12.1 |  |
| P33 | 103.5 | 80.6 | 15 | 30.4 | 1.4 |  |
| P34 | 119 | 78.3 | 54 | 34.1 | 10 |  |
| P34 | 119.2 | 75.5 | 43.8 | 31.1 | 10 |  |
| P34 | 139.2 | 76.6 | 65.3 | 22.2 | 13.6 |  |
| P36 | 177.4 | 169.7 | 140.2 | 84.9 | 51.8 |  |
| P36 | 183.4 | . | 142.3 | 111.9 | 64.3 |  |
| P36 | 192.5 | 184 | 143.7 | 97.8 | 80 |  |
| *P42 | 61.9 | 45.4 | 35.4 | 24.4 | 1.7 |  |
| *P42 | 56.2 | 43.5 | 29.5 | 17.9 | 10.1 |  |
| *P42 | 63.7 | 49.6 | 32.1 | 21.7 | 10.4 |  |
| *P51 | 98.7 | 92.3 | 70.2 | 47 | 17.5 |  |
| *P51 | 100.2 | 99.6 | 71.8 | 50.4 | 4.6 |  |
| *P51 | 90.9 | 93.4 | 55.7 | 39.7 | . |  |

**

**

**Suppl. Figure 11: Typical fluorescence microscopy images from primary oral keratinocytes stained for gammaH2AX and 53BP1 foci. Colocalized nuclear foci were counted as DNA double strand breaks.**
