# Supplementary material for: Biopsy-derived oral keratinocytes – A model to potentially test for oral mucosa radiation sensitivity
Source: Clin Transl Radiat Oncol. 2022 Mar 16;34:51–6. doi: 10.1016/j.ctro.2022.03.007 (PMC8956846; doi:10.1016/j.ctro.2022.03.007)
Supplement: Supplementary data 2 [file mmc2.pdf]

Raw data on double strand repair analysis (colocalized gammaH2AX/53BP1 foci)

Estimated increase in foci was calculated using the following R code:

```
model <- glm(foci/cell~dose, data = sub_DF, family = quasipoisson(link = "identity"))
estimated_increase_in_foci_numbers <- coef(model)[2]
```

Technically this is a calculation of the difference between 6 and 0 Gy using a generalized linear model with a quasipoisson error distribution

Volunteer	Dose [Gy]	Time[h]	Coloc. foci/cell	Dose [Gy]	Time[h]	Coloc. foci/cell	Dose [Gy]	Time[h]	Coloc. foci/cell	Dose [Gy]	Time[h]	Coloc. foci/cell
P30	0	24	0	6	24	2	0	96	1	6	96	0
P30	0	24	0	6	24	4	0	96	1	6	96	3
P30	0	24	1	6	24	8	0	96	0	6	96	4
P30	0	24	0	6	24	6	0	96	2	6	96	0
P30	0	24	1	6	24	3	0	96	0	6	96	2
P30	0	24	1	6	24	3	0	96	0	6	96	1
P30	0	24	2	6	24	4	0	96	0	6	96	0
P30	0	24	0	6	24	3	0	96	2	6	96	3
P30	0	24	0	6	24	3	0	96	2	6	96	1
P30	0	24	0	6	24	6	0	96	3	6	96	0
P30	0	24	0	6	24	4	0	96	2	6	96	0
P30	0	24	0	6	24	0	0	96	0	6	96	0
P30	0	24	0	6	24	12	0	96	1	6	96	0
P30	0	24	6	6	24	5	0	96	0	6	96	3
P30	0	24	0	6	24	2	0	96	0	6	96	0
P30	0	24	0	6	24	6	0	96	1	6	96	1
P30	0	24	0	6	24	0	0	96	0	6	96	2
P30	0	24	2	6	24	3	0	96	0	6	96	5
P30	0	24	2	6	24	3	0	96	0	6	96	2
P30	0	24	0	6	24	5	0	96	1	6	96	0
P30	0	24	0	6	24	2	0	96	0	6	96	0
P30	0	24	0	6	24	1	0	96	0	6	96	0
P30	0	24	1	6	24	0	0	96	1	6	96	0
P30	0	24	1	6	24	9	0	96	2	6	96	1
P30	0	24	0	6	24	5	0	96	0	6	96	2
P30	0	24	0	6	24	1	0	96	0	6	96	0
P30	0	24	0	6	24	3	0	96	0	6	96	3
P30	0	24	1	6	24	2	0	96	1	6	96	1
P30	0	24	0	6	24	1	0	96	2	6	96	1
P30	0	24	0	6	24	5	0	96	0	6	96	0
P30	0	24	0	6	24	2	0	96	0	6	96	0
P30	0	24	0	6	24	2	0	96	1	6	96	1
P30	0	24	0	6	24	1	0	96	0	6	96	1
P30	0	24	0	6	24	8	0	96	1	6	96	2
P30	0	24	0	6	24	4	0	96	0	6	96	0
P30	0	24	0	6	24	7	0	96	4	6	96	0
P30	0	24	0	6	24	8	0	96	1	6	96	0
P30	0	24	0	6	24	4	0	96	0	6	96	0
P30	0	24	1	6	24	1	0	96	0	6	96	2
P30	0	24	0	6	24	0	0	96	3	6	96	0
P30	0	24	0	6	24	3	0	96	1	6	96	4
P30	0	24	1	6	24	1	0	96	2	6	96	0
P30	0	24	1	6	24	1	0	96	0	6	96	0
P30	0	24	0	6	24	3	0	96	0	6	96	0
P30	0	24	0	6	24	5	0	96	0	6	96	3
P30	0	24	0	6	24	10	0	96	0	6	96	2
P30	0	24	3	6	24	3	0	96	2	6	96	0
P30	0	24	0	6	24	8	0	96	0	6	96	0
P30	0	24	2	6	24	0	0	96	1	6	96	1
P30	0	24	0	6	24	2	0	96	1	6	96	0
Mean			0.520			3.680			0.780			1.020

Volunteer	Dose [Gy]	Time[h]	Coloc. foci/cell	Dose [Gy]	Time[h]	Coloc. foci/cell	Dose [Gy]	Time[h]	Coloc. foci/cell	Dose [Gy]	Time[h]	Coloc. foci/cell
P31	0	24	0	6	24	3	0	96	2	6	96	1
P31	0	24	0	6	24	10	0	96	0	6	96	3
P31	0	24	0	6	24	2	0	96	0	6	96	2
P31	0	24	0	6	24	1	0	96	1	6	96	0
P31	0	24	0	6	24	5	0	96	0	6	96	6
P31	0	24	1	6	24	2	0	96	0	6	96	1
P31	0	24	0	6	24	3	0	96	0	6	96	1
P31	0	24	0	6	24	4	0	96	0	6	96	0
P31	0	24	2	6	24	4	0	96	0	6	96	0
P31	0	24	0	6	24	0	0	96	0	6	96	0
P31	0	24	0	6	24	4	0	96	1	6	96	3
P31	0	24	0	6	24	1	0	96	0	6	96	1
P31	0	24	0	6	24	2	0	96	2	6	96	2
P31	0	24	1	6	24	8	0	96	0	6	96	0
P31	0	24	0	6	24	4	0	96	0	6	96	1
P31	0	24	0	6	24	3	0	96	2	6	96	3
P31	0	24	1	6	24	5	0	96	0	6	96	0
P31	0	24	0	6	24	3	0	96	0	6	96	0
P31	0	24	0	6	24	0	0	96	0	6	96	1
P31	0	24	0	6	24	0	0	96	2	6	96	1
P31	0	24	0	6	24	0	0	96	0	6	96	2
P31	0	24	0	6	24	1	0	96	0	6	96	2
P31	0	24	1	6	24	7	0	96	0	6	96	4
P31	0	24	2	6	24	2	0	96	3	6	96	4
P31	0	24	0	6	24	7	0	96	0	6	96	0
P31	0	24	1	6	24	4	0	96	0	6	96	1
P31	0	24	0	6	24	1	0	96	1	6	96	3
P31	0	24	0	6	24	0	0	96	0	6	96	1
P31	0	24	0	6	24	0	0	96	0	6	96	1
P31	0	24	0	6	24	0	0	96	0	6	96	0
P31	0	24	1	6	24	9	0	96	0	6	96	6
P31	0	24	3	6	24	1	0	96	1	6	96	3
P31	0	24	0	6	24	0	0	96	2	6	96	0
P31	0	24	0	6	24	5	0	96	0	6	96	0
P31	0	24	0	6	24	1	0	96	1	6	96	2
P31	0	24	1	6	24	9	0	96	1	6	96	0
P31	0	24	0	6	24	0	0	96	1	6	96	0
P31	0	24	0	6	24	7	0	96	0	6	96	1
P31	0	24	0	6	24	7	0	96	1	6	96	0

P31	0	24	0	6	24	3	0	96	0	6	96	0
P31	0	24	1	6	24	4	0	96	2	6	96	4
P31	0	24	0	6	24	0	0	96	1	6	96	6
P31	0	24	0	6	24	2	0	96	0	6	96	0
P31	0	24	0	6	24	7	0	96	2	6	96	2
P31	0	24	0	6	24	8	0	96	0	6	96	0
P31	0	24	0	6	24	3	0	96	0	6	96	0
P31	0	24	0	6	24	5	0	96	0	6	96	0
P31	0	24	0	6	24	0	0	96	0	6	96	5
P31	0	24	0	6	24	4	0	96	3	6	96	1
P31	0	24	0	6	24	0	0	96	0	6	96	3
Mean			0.300			3.220			0.580			1.540

Volunteer	Dose [Gy]	Time[h]	Coloc. foci/cell	Dose [Gy]	Time[h]	Coloc. foci/cell	Dose [Gy]	Time[h]	Coloc. foci/cell	Dose [Gy]	Time[h]	Coloc. foci/cell
P49	0	24	0	6	24	0	0	96	0	6	96	1
P49	0	24	0	6	24	3	0	96	0	6	96	3
P49	0	24	0	6	24	0	0	96	2	6	96	11
P49	0	24	0	6	24	5	0	96	0	6	96	0
P49	0	24	0	6	24	0	0	96	0	6	96	0
P49	0	24	0	6	24	1	0	96	0	6	96	2
P49	0	24	0	6	24	0	0	96	0	6	96	7
P49	0	24	0	6	24	0	0	96	0	6	96	1
P49	0	24	0	6	24	3	0	96	4	6	96	2
P49	0	24	0	6	24	0	0	96	0	6	96	0
P49	0	24	0	6	24	5	0	96	0	6	96	0
P49	0	24	0	6	24	5	0	96	0	6	96	0
P49	0	24	0	6	24	3	0	96	0	6	96	0
P49	0	24	2	6	24	0	0	96	0	6	96	0
P49	0	24	1	6	24	2	0	96	0	6	96	2
P49	0	24	1	6	24	0	0	96	1	6	96	0
P49	0	24	3	6	24	5	0	96	0			
P49	0	24	0	6	24	2	0	96	1			
P49	0	24	0	6	24	3	0	96	0			
P49	0	24	2	6	24	0	0	96	0			
P49	0	24	3	6	24	3	0	96	0			
P49	0	24	0	6	24	3	0	96	0			
P49	0	24	0	6	24	4	0	96	0			
P49	0	24	0	6	24	1	0	96	0			
P49	0	24	0	6	24	2	0	96	0			
P49	0	24	1	6	24	5	0	96	0			
P49	0	24	2	6	24	10	0	96	0			
P49	0	24	0	6	24	1	0	96	1			
P49	0	24	0	6	24	1	0	96	0			
P49	0	24	1	6	24	5	0	96	2			
P49	0	24	0	6	24	3	0	96	0			
P49	0	24	0	6	24	4	0	96	1			
P49	0	24	3	6	24	7	0	96	1			
P49	0	24	2	6	24	2	0	96	0			
P49	0	24	0	6	24	4	0	96	0			
P49	0	24	0	6	24	30	0	96	0			
P49	0	24	2	6	24	30	0	96	0			
P49	0	24	1	6	24	4	0	96	0			
P49	0	24	2	6	24	3	0	96	0			
P49	0	24	0	6	24	3	0	96	0			
P49	0	24	3	6	24	8	0	96	0			
P49	0	24	3	6	24	1	0	96	0			
P49	0	24	2	6	24	2	0	96	0			
P49	0	24	0	6	24	8	0	96	0			
P49	0	24	0	6	24	0	0	96	0			
P49	0	24	0	6	24	3	0	96	0			
P49	0	24	0	6	24	1	0	96	0			
P49	0	24	0	6	24	2	0	96	1			
P49	0	24	1	6	24	2	0	96	0			
P49	0	24	0	6	24	5	0	96	0			
P49	0	24	0	6	24	16	0	96	3			
P49	0	24	0	6	24	1	0	96	0			
P49	0	24	0	6	24	1	0	96	0			
P49	0	24	0	6	24	8	0	96	0			
P49	0	24	0	6	24	5	0	96	0			
P49	0	24	2	6	24	3	0	96	3			
P49	0	24	0	6	24	5	0	96	0			
P49	0	24	0	6	24	2	0	96	2			
P49	0	24	1	6	24	2	0	96	2			
P49	0	24	0	6	24	2	0	96	0			
P49	0	24	0	6	24	3	0	96	0			
P49	0	24	0	6	24	6	0	96	0			
P49	0	24	0	6	24	5	0	96	0			
P49	0	24	0	6	24	1	0	96	2			
P49	0	24	0	6	24	1	0	96	0			
P49	0	24	3	6	24	2	0	96	0			
P49	0	24	0	6	24	2	0	96	1			
P49	0	24	0	6	24	3	0	96	0			
P49	0	24	0	6	24	12	0	96	1			
P49	0	24	0	6	24	8	0	96	0			
P49	0	24	1	6	24	7	0	96	0			
P49	0	24	2	6	24	6	0	96	1			
P49	0	24	0	6	24	5	0	96	0			
P49	0	24	0	6	24	2	0	96	1			
P49	0	24	0	6	24	4	0	96	0			
P49	0	24	0	6	24	2	0	96	0			
P49	0	24	0	6	24	3	0	96	2			
P49	0	24	0	6	24	2	0	96	0			
P49	0	24	0	6	24	2	0	96	0			
P49	0	24	0	6	24	2	0	96	0			
P49	0	24	0	6	24	2	0	96	0			
P49	0	24	0	6	24	5	0	96	0			
P49	0	24	1	6	24	3	0	96	0			
P49	0	24	0	6	24	0	0	96	0			
P49	0	24	0	6	24	0	0	96	0			
P49	0	24	0	6	24	1	0	96	0			

P49	0	24	0	6	24	1	0	96	0			
P49	0	24	0	6	24	0	0	96	3			
P49	0	24	0	6	24	0	0	96	0			
P49	0	24	1	6	24	0	0	96	0			
P49	0	24	0	6	24	2	0	96	0			
P49	0	24	0	6	24	3	0	96	0			
P49	0	24	0	6	24	2	0	96	0			
P49	0	24	0	6	24	0	0	96	0			
P49	0	24	0	6	24	1	0	96	0			
P49	0	24	0	6	24	2	0	96	0			
P49	0	24	0	6	24	4	0	96	2			
P49	0	24	0	6	24	2	0	96	2			
P49	0	24	0	6	24	3	0	96	6			
P49	0	24	0	6	24	2	0	96	0			
P49	0	24	0	6	24	3	0	96	0			
P49	0	24	0	6	24	1	0	96	0			
P49	0	24	0	6	24	0	0	96	0			
P49	0	24	0	6	24	3	0	96	0			
P49	0	24	0	6	24	6	0	96	0			
P49	0	24	0	6	24	0	0	96	0			
P49	0	24	1	6	24	7	0	96	0			
P49	0	24	0	6	24	5	0	96	2			
P49	0	24	0	6	24	5	0	96	0			
P49	0	24	2	6	24	3	0	96	0			
P49	0	24	2	6	24	7						
P49	0	24	2	6	24	4						
P49	0	24	0	6	24	0						
P49	0	24	0	6	24	2						
P49	0	24	0	6	24	1						
P49	0	24	0	6	24	10						
P49	0	24	0	6	24	0						
P49	0	24	0	6	24	1						
P49	0	24	0	6	24	3						
P49	0	24	0	6	24	4						
P49	0	24	0	6	24	3						
P49	0	24	0	6	24	0						
P49	0	24	0	6	24	3						
P49	0	24	0	6	24	4						
P49	0	24	0	6	24	3						
P49	0	24	0	6	24	2						
P49	0	24	0	6	24	2						
P49	0	24	0	6	24	10						
P49	0	24	0	6	24	9						
P49	0	24	0	6	24	0						
P49	0	24	0	6	24	0						
P49	0	24	0	6	24	3						
P49	0	24	0	6	24	2						
P49	0	24	2	6	24	4						
P49	0	24	0	6	24	7						
P49	0	24	2	6	24	0						
P49	0	24	1	6	24	2						
P49	0	24	0	6	24	5						
P49	0	24	0	6	24	5						
P49	0	24	0	6	24	4						
P49	0	24	1	6	24	7						
P49	0	24	1	6	24	5						
P49	0	24	1	6	24	2						
P49	0	24	3	6	24	0						
P49	0	24	0	6	24	2						
P49	0	24	2	6	24	3						
P49	0	24	3	6	24	4						
P49	0	24	0	6	24	6						
P49	0	24	0	6	24	9						
P49	0	24	0	6	24	30						
P49	0	24	0	6	24	4						
P49	0	24	0									
P49	0	24	0									
P49	0	24	0									
P49	0	24	0									
P49	0	24	3									
P49	0	24	0									
P49	0	24	0									
P49	0	24	0									
P49	0	24	0									
P49	0	24	0									
P49	0	24	0									
P49	0	24	0									
P49	0	24	0									
P49	0	24	0									
P49	0	24	0									
P49	0	24	0									
Mean			0.431			3.675			0.427			1.813

Volunteer	Dose [Gy]	Time[h]	Coloc. foci/cell	Dose [Gy]	Time[h]	Coloc. foci/cell	Dose [Gy]	Time[h]	Coloc. foci/cell	Dose [Gy]	Time[h]	Coloc. foci/cell
P51	0	24	0	6	24	0	0	96	9	6	96	1
P51	0	24	0	6	24	6	0	96	0	6	96	0
P51	0	24	0	6	24	21	0	96	4	6	96	0
P51	0	24	1	6	24	0	0	96	0	6	96	2
P51	0	24	0	6	24	2	0	96	0	6	96	1
P51	0	24	0	6	24	2	0	96	0	6	96	0
P51	0	24	0	6	24	1	0	96	0	6	96	0
P51	0	24	0	6	24	1	0	96	0	6	96	0
P51	0	24	0	6	24	3	0	96	1	6	96	3
P51	0	24	0	6	24	3	0	96	0	6	96	3
P51	0	24	0	6	24	10	0	96	0	6	96	3
P51	0	24	0	6	24	5	0	96	0	6	96	0
P51	0	24	2	6	24	2	0	96	0	6	96	2
P51	0	24	2	6	24	2	0	96	0	6	96	0
P51	0	24	2	6	24	0	0	96	0	6	96	2
P51	0	24	2	6	24	0	0	96	0	6	96	2

P51	0	24	2
P51	0	24	1
P51	0	24	0
P51	0	24	2
P51	0	24	0
P51	0	24	1
P51	0	24	0
P51	0	24	0
P51	0	24	0
P51	0	24	0
P51	0	24	0
P51	0	24	3
P51	0	24	0
P51	0	24	0
P51	0	24	0
P51	0	24	0
P51	0	24	0
P51	0	24	0
P51	0	24	5
P51	0	24	2
P51	0	24	0
P51	0	24	0
P51	0	24	0
P51	0	24	2
P51	0	24	1
P51	0	24	0
P51	0	24	0
P51	0	24	0
P51	0	24	10
P51	0	24	0
P51	0	24	0
P51	0	24	1
P51	0	24	1
P51	0	24	1
P51	0	24	0
P51	0	24	0
P51	0	24	1
P51	0	24	0
P51	0	24	0
P51	0	24	0
P51	0	24	1
P51	0	24	1
P51	0	24	2
P51	0	24	4
P51	0	24	0
P51	0	24	0
P51	0	24	2
P51	0	24	0
P51	0	24	0
P51	0	24	3
P51	0	24	0
P51	0	24	0
P51	0	24	0
P51	0	24	0
P51	0	24	0
P51	0	24	0
P51	0	24	0
P51	0	24	0
P51	0	24	0
P51	0	24	0
P51	0	24	0
P51	0	24	2
P51	0	24	2
P51	0	24	0
P51			
P51			
P51			
P51			
P51			
P51			
P51			
P51			
P51			
P51			
P51			
P51			
P51			
P51			
P51			
P51			
P51			
P51			
P51			
P51			
P51			
P51			
P51			
P51			
P51			
P51			
P51			
P51			
P51			
P51			
P51			
P51			
P51			
P51			
P51			
P51			

6	24	0
6	24	0
6	24	9
6	24	0
6	24	4
6	24	1
6	24	3
6	24	1
6	24	3
6	24	2
6	24	5
6	24	0
6	24	2
6	24	0
6	24	0
6	24	9
6	24	3
6	24	2
6	24	5
6	24	1
6	24	0
6	24	3
6	24	2
6	24	2
6	24	0
6	24	0
6	24	12
6	24	0
6	24	0
6	24	4
6	24	2
6	24	3
6	24	3
6	24	0
6	24	1
6	24	2
6	24	4
6	24	11
6	24	3
6	24	0
6	24	0
6	24	0
6	24	0
6	24	5
6	24	5
6	24	2

0	96	0
0	96	0
0	96	0
0	96	0
0	96	0
0	96	0
0	96	1
0	96	1
0	96	2
0	96	0
0	96	2
0	96	0
0	96	0
0	96	0
0	96	0
0	96	0
0	96	0
0	96	0
0	96	0
0	96	0
0	96	3
0	96	0
0	96	1
0	96	0
0	96	0
0	96	1
0	96	0
0	96	0
0	96	2
0	96	0
0	96	1
0	96	2
0	96	0
0	96	0
0	96	2
0	96	0
0	96	0
0	96	2
0	96	0
0	96	0
0	96	0
0	96	0
0	96	0
0	96	0
0	96	0
0	96	0
0	96	0
0	96	1
0	96	0
0	96	0
0	96	0
0	96	1
0	96	2
0	96	0
0	96	0
0	96	0
0	96	0
0	96	0
0	96	0
0	96	0
0	96	0
0	96	0
0	96	0
0	96	0
0	96	0
0	96	0
0	96	0
0	96	0
0	96	0
0	96	0
0	96	0
0	96	0

6	96	0
6	96	0
6	96	2
6	96	0
6	96	0
6	96	0
6	96	1
6	96	0
6	96	3
6	96	5
6	96	2
6	96	0
6	96	4
6	96	3
6	96	3
6	96	2
6	96	0
6	96	0
6	96	0
6	96	5
6	96	3
6	96	3
6	96	3
6	96	2
6	96	0
6	96	2
6	96	1
6	96	0
6	96	0
6	96	1
6	96	1
6	96	0
6	96	2
6	96	2
6	96	0
6	96	0
6	96	0
6	96	0
6	96	0
6	96	4
6	96	0
6	96	0
6	96	12
6	96	5
6	96	4
6	96	2
6	96	2
6	96	1
6	96	0
6	96	0
6	96	0
6	96	0
6	96	5
6	96	1
6	96	0
6	96	0
6	96	0
6	96	0
6	96	0
6	96	0
6	96	0
6	96	0
6	96	5
6	96	0
6	96	0
6	96	0
6	96	0
6	96	0
6	96	4
6	96	1
6	96	1
6	96	2
6	96	0
6	96	0
6	96	0
6	96	0
6	96	0
6	96	0
6	96	2
6	96	0
6	96	1
6	96	0
6	96	2
6	96	0
6	96	7
6	96	1
6	96	0
6	96	1
6	96	0
6	96	1
6	96	1
6	96	3
6	96	2
6	96	0
6	96	2
6	96	1
6	96	2
6	96	0
6	96	3
6	96	2

P51										6	96	0
P51										6	96	1
P51										6	96	0
P51										6	96	0
P51										6	96	2
P51										6	96	1
P51										6	96	1
P51										6	96	0
P51										6	96	4
P51										6	96	0
P51										6	96	0
P51										6	96	0
P51										6	96	0
P51										6	96	0
P51										6	96	0
P51										6	96	0
P51										6	96	0
P51										6	96	0
P51										6	96	2
P51										6	96	2
P51										6	96	1
P51										6	96	0
P51										6	96	2
P51										6	96	1
P51										6	96	4
P51										6	96	0
P51										6	96	2
P51										6	96	0
P51										6	96	1
P51										6	96	0
P51										6	96	0
P51										6	96	0
P51										6	96	0
P51										6	96	1
P51										6	96	6
P51										6	96	0
P51										6	96	0
P51										6	96	0
P51										6	96	0
Mean			0.738			2.774			0.437			1.161

Volunteer	Dose [Gy]	Time[h]	Coloc. foci/cell	Dose [Gy]	Time[h]	Coloc. foci/cell	Dose [Gy]	Time[h]	Coloc. foci/cell	Dose [Gy]	Time[h]	Coloc. foci/cell
P52	0	24	0	6	24	32	0	96	0	6	96	8
P52	0	24	0	6	24	11	0	96	3	6	96	3
P52	0	24	0	6	24	48	0	96	2	6	96	0
P52	0	24	2	6	24	8	0	96	0	6	96	0
P52	0	24	2	6	24	3	0	96	0	6	96	0
P52	0	24	2	6	24	6	0	96	2	6	96	0
P52	0	24	1	6	24	12	0	96	4	6	96	0
P52	0	24	0	6	24	0	0	96	0	6	96	1
P52	0	24	0	6	24	18	0	96	0	6	96	1
P52	0	24	3	6	24	14	0	96	1	6	96	0
P52	0	24	0	6	24	0	0	96	0	6	96	0
P52	0	24	0	6	24	41	0	96	3	6	96	0
P52	0	24	0	6	24	16	0	96	0	6	96	1
P52	0	24	2	6	24	1	0	96	2	6	96	0
P52	0	24	0	6	24	15	0	96	0	6	96	0
P52	0	24	0	6	24	33	0	96	1	6	96	0
P52	0	24	0	6	24	5	0	96	3	6	96	0
P52	0	24	2	6	24	0	0	96	2	6	96	0
P52	0	24	0	6	24	0	0	96	0	6	96	2
P52	0	24	0	6	24	0	0	96	2	6	96	2
P52	0	24	0	6	24	0	0	96	0	6	96	0
P52	0	24	0	6	24	0	0	96	0	6	96	0
P52	0	24	0	6	24	0	0	96	0	6	96	0
P52	0	24	0	6	24	5	0	96	0	6	96	0
P52	0	24	0	6	24	0	0	96	0	6	96	0
P52	0	24	0	6	24	4	0	96	0	6	96	0
P52	0	24	0	6	24	0	0	96	0	6	96	2
P52	0	24	0	6	24	0	0	96	0	6	96	1
P52	0	24	0	6	24	0	0	96	0	6	96	0
P52	0	24	0	6	24	23	0	96	5	6	96	1
P52	0	24	4	6	24	0	0	96	3	6	96	0
P52	0	24	5				0	96	3	6	96	0
P52	0	24	0				0	96	0	6	96	0
P52	0	24	0				0	96	0	6	96	0
P52	0	24	0				0	96	0	6	96	0
P52	0	24	2				0	96	0	6	96	0
P52							0	96	0	6	96	0
P52							0	96	0	6	96	0
P52							0	96	0	6	96	0
P52							0	96	0	6	96	0
P52							0	96	0	6	96	0
P52							0	96	0	6	96	0
P52							0	96	0	6	96	0
P52							0	96	0	6	96	2
P52							0	96	0	6	96	0
P52							0	96	0	6	96	0
P52							0	96	0	6	96	0
P52							0	96	5	6	96	0
P52							0	96	3	6	96	0
P52							0	96	0	6	96	0
P52							0	96	0	6	96	0
P52							0	96	0	6	96	0
P52							0	96	0	6	96	0
P52							0	96	0	6	96	0
P52							0	96	0	6	96	0
P52							0	96	0	6	96	2
P52							0	96	0	6	96	2
P52							0	96	3	6	96	0

P52							0	96	0	6	96	0
P52							0	96	1	6	96	0
P52							0	96	5	6	96	0
P52							0	96	0	6	96	0
P52							0	96	0	6	96	0
P52							0	96	0	6	96	2
P52							0	96	0	6	96	0
P52							0	96	0	6	96	1
P52							0	96	0	6	96	0
P52							0	96	0	6	96	0
P52							0	96	3			
P52							0	96	2			
Mean			0.694			9.516			0.829			0.456

Volunteer	Dose [Gy]	Time[h]	Coloc. foci/cell	Dose [Gy]	Time[h]	Coloc. foci/cell	Dose [Gy]	Time[h]	Coloc. foci/cell	Dose [Gy]	Time[h]	Coloc. foci/cell
P53	0	24	0	6	24	0	0	96	13	6	96	0
P53	0	24	3	6	24	0	0	96	0	6	96	0
P53	0	24	0	6	24	6	0	96	0	6	96	4
P53	0	24	2	6	24	0	0	96	0	6	96	0
P53	0	24	2	6	24	0	0	96	0	6	96	0
P53	0	24	0	6	24	0	0	96	0	6	96	0
P53	0	24	1	6	24	6	0	96	0	6	96	0
P53	0	24	0	6	24	9	0	96	1	6	96	0
P53	0	24	0	6	24	2	0	96	1	6	96	0
P53	0	24	0	6	24	5	0	96	0	6	96	0
P53	0	24	0	6	24	3	0	96	0	6	96	0
P53	0	24	3	6	24	3	0	96	0	6	96	0
P53	0	24	0	6	24	3	0	96	2	6	96	0
P53	0	24	0	6	24	0	0	96	0	6	96	5
P53	0	24	0	6	24	3	0	96	0	6	96	0
P53	0	24	0	6	24	5	0	96	0	6	96	3
P53	0	24	0	6	24	26	0	96	0	6	96	5
P53	0	24	0	6	24	0	0	96	0	6	96	5
P53	0	24	2	6	24	0	0	96	3	6	96	5
P53	0	24	0	6	24	0	0	96	0	6	96	0
P53	0	24	0	6	24	0	0	96	0	6	96	0
P53	0	24	2	6	24	0	0	96	0	6	96	0
P53	0	24	0	6	24	0	0	96	0	6	96	1
P53	0	24	0	6	24	0	0	96	0	6	96	3
P53	0	24	0	6	24	3	0	96	0	6	96	0
P53	0	24	0	6	24	0	0	96	5	6	96	0
P53	0	24	0	6	24	4	0	96	0	6	96	2
P53	0	24	0	6	24	0	0	96	1	6	96	0
P53	0	24	3	6	24	2	0	96	0	6	96	0
P53	0	24	0	6	24	5	0	96	0	6	96	0
P53	0	24	0	6	24	2	0	96	0	6	96	0
P53	0	24	0	6	24	0	0	96	3	6	96	0
P53	0	24	0	6	24	2	0	96	2	6	96	0
P53	0	24	0	6	24	2	0	96	4	6	96	0
P53	0	24	0	6	24	2	0	96	0	6	96	0
P53	0	24	0	6	24	9	0	96	0	6	96	5
P53	0	24	0				0	96	0	6	96	0
P53	0	24	0				0	96	5	6	96	0
P53	0	24	0				0	96	0	6	96	4
P53	0	24	0				0	96	3	6	96	0
P53	0	24	0				0	96	0	6	96	0
P53	0	24	0							6	96	0
P53	0	24	0							6	96	1
P53	0	24	0							6	96	0
P53	0	24	2							6	96	3
P53	0	24	0							6	96	0
P53	0	24	0							6	96	0
P53	0	24	0							6	96	0
P53	0	24	0							6	96	0
P53	0	24	0							6	96	5
P53	0	24	3							6	96	8
P53	0	24	2							6	96	7
P53	0	24	0							6	96	0
P53	0	24	0							6	96	0
P53	0	24	0							6	96	0
P53	0	24	0							6	96	3
P53	0	24	0							6	96	5
P53	0	24	0							6	96	5
P53	0	24	0							6	96	2
P53	0	24	0							6	96	0
P53										6	96	0
P53										6	96	0
P53										6	96	2
P53										6	96	3
P53										6	96	0
P53										6	96	2
Mean			0.417			2.833			1.049			1.333
