# Supplementary material for: Biopsy-derived oral keratinocytes – A model to potentially test for oral mucosa radiation sensitivity
Source: Clin Transl Radiat Oncol. 2022 Mar 16;34:51–6. doi: 10.1016/j.ctro.2022.03.007 (PMC8956846; doi:10.1016/j.ctro.2022.03.007)
Supplement: Supplementary data 3 [file mmc3.pdf]

Pearson correlation coefficient between area and logKBE

```
cor(Area, logKBE, use = "complete.obs", method = "pearson")
```

```
## [1] 0.4062589
```

Linear mixed models

Model 1: log(KBE) on Dose [Gy]: Strong effect of dose

```
model1 <- lmer(logKBE ~ Gy + (1|Donor))
summary(model1)
```

```
## Linear mixed model fit by REML ['lmerMod']
## Formula: logKBE ~ Gy + (1 | Donor)
##
## REML criterion at convergence: 780
##
## Scaled residuals:
##     Min      1Q  Median      3Q     Max
## -5.5880 -0.0343  0.1657  0.3353  1.0775
##
## Random effects:
##  Groups   Name        Variance Std.Dev.
##  Donor    (Intercept)  2.109   1.452
##  Residual             10.944   3.308
## Number of obs: 146, groups:  Donor, 15
##
## Fixed effects:
##             Estimate Std. Error t value
## (Intercept) -1.72353    0.60430  -2.852
## Gy          -0.74278    0.09776  -7.598
##
## Correlation of Fixed Effects:
##    (Intr)
## Gy -0.640
```
