# Supplementary material for: Biopsy-derived oral keratinocytes – A model to potentially test for oral mucosa radiation sensitivity
Source: Clin Transl Radiat Oncol. 2022 Mar 16;34:51–6. doi: 10.1016/j.ctro.2022.03.007 (PMC8956846; doi:10.1016/j.ctro.2022.03.007)
Supplement: Supplementary data 4 [file mmc4.pdf]

# Model 4: log(KBE) on Area, Dose [Gy] and Group: No effect of group

```
model4 <- lmer(logKBE ~ Gy + (1|Donor) + Area + Group)
summary(model4)
```

```
## Linear mixed model fit by REML ['lmerMod']
## Formula: logKBE ~ Gy + (1 | Donor) + Area + Group
##
## REML criterion at convergence: 781.7
##
## Scaled residuals:
##     Min      1Q  Median      3Q     Max
## -5.5181 -0.0248  0.1610  0.3253  1.0515
##
## Random effects:
##  Groups   Name        Variance Std.Dev.
##  Donor    (Intercept)  2.246   1.499
##  Residual             11.124   3.335
## Number of obs: 145, groups:  Donor, 15
##
## Fixed effects:
##               Estimate Std. Error t value
## (Intercept)  -2.099400   1.337636  -1.569
## Gy           -0.683467   0.167267  -4.086
## Area          0.003336   0.007589   0.440
## GroupPatient -0.377593   1.009511  -0.374
##
## Correlation of Fixed Effects:
##             (Intr) Gy     Area
## Gy          -0.839
## Area        -0.822  0.807
## GroupPatint -0.050 -0.254 -0.324
```

```
confint(model4)
```

```
##                    2.5 %      97.5 %
## .sig01        0.52021179  2.32291739
## .sigma        2.94955036  3.76608561
## (Intercept)  -4.75439388  0.47853593
## Gy           -1.00204287 -0.34233498
## Area         -0.01128937  0.01884944
## GroupPatient -2.29484872  1.57838145
```
